# CladeOScope-GSA: Revealing Evolutionary Associations Across Gene Sets

**DOI:** 10.3390/ijms27031457

**Published:** 2026-02-01

**Authors:** Maya Braun, Idit Bloch, Dana Sherill-Rofe, Christina Canavati, Elad Sharon, Yuval Tabach

**Affiliations:** Department of Developmental Biology and Cancer Research, Institute for Medical Research Israel-Canada, Hebrew University of Jerusalem, Jerusalem 9112102, Israel; maya.braun@mail.huji.ac.il (M.B.); iditblochlab@gmail.com (I.B.); sherill@hadassah.org.il (D.S.-R.); christin.canavati@mail.huji.ac.il (C.C.); elad.sharon@mail.huji.ac.il (E.S.)

**Keywords:** phylogenetic profiling, comparative genomics, gene set analysis, functional genomics

## Abstract

Deciphering gene and protein functions and interactions remains a core challenge in biology and medicine. Gene set analysis and multi-omics tools are widely used to interpret gene lists; however, they often overlook shared evolutionary patterns among genes. These conservation and loss patterns, shaped by billions of years of evolutionary pressure, can uncover co-evolutionary signals within gene sets, yet they remain frequently underexplored. In this study, we apply normalized phylogenetic profiling (NPP) across 1905 eukaryotic species and introduce CladeOScope-GSA, a tool for analyzing user-defined gene sets. CladeOScope-GSA uncovers common signatures of conservation, revealing whether a gene set evolves as a cohesive unit or as distinct co-evolving submodules. By tracing gene set origins, diversification, and shared evolutionary histories, the tool identifies the structural organization and key components of gene networks, exposing functional similarities, phenotypic associations, and broader biological relationships. We demonstrate its utility through two well-characterized cases: the porphyria-related pathway and the dynein gene family. In both, CladeOScope-GSA recapitulates known functional substructures and uncovers previously unrecognized evolutionary insights, underscoring its value for advancing our understanding of gene function and pathway evolution on a broad scale.

## 1. Introduction

Advances in high-throughput sequencing and other omics technologies have created an unprecedented volume of genomic and proteomic data. These experiments often yield gene lists whose genetic or epigenetic alterations are associated with disease pathophysiology, phenotypic traits, responses to stimuli, and other molecular processes. Interpreting these gene lists by identifying their shared functions, interactions, and regulatory mechanisms, is essential for translating raw omics data into meaningful biomedical insights.

Pathway databases such as PathBank [1], KEGG [2], and Reactome [3], along with various bioinformatic tools, have been developed to facilitate the analysis of gene and protein sets. These resources help researchers interpret experimental data by mapping genes to known biological pathways and systems, uncovering the molecular mechanisms underlying specific experimental contexts. Gene co-expression analysis identifies gene modules that share regulatory mechanisms [4]. Other approaches integrate pathway topology and gene interaction data to highlight structural features such as hub genes, highly connected genes that may play key regulatory roles [5]. The Gene Ontology (GO) project, launched in 1998, is a foundational bioinformatics initiative that standardizes the description of gene and gene product functions across species, providing a unified vocabulary to support functional genomics research [6,7]. These integrative analyses have transformed our understanding of biological systems by offering standardized vocabularies and frameworks to describe gene functions and interactions within cells and organisms [6].

Over the course of eukaryotic evolution, biological pathways have continuously emerged and diversified, undergoing processes of rewiring, optimization, or even complete loss. These evolutionary dynamics not only shaped the molecular architecture of pathways but also determined how organisms adapt, survive, and respond to changing ecological and environmental pressures [8,9]. Nevertheless, most current gene set analysis methods do not incorporate evolutionary information, and therefore miss key insights about gene function conservation and divergence across species [10]. As a result, information such as which genes in a set are conserved or lost in particular species, or what the evolutionary origin of these genes is, is not provided. Most importantly, it remains unclear whether gene sets display common evolutionary patterns or consistent conservation trends across the tree of life.

Phylogenetic profiling (PP) is a complementary approach that addresses many of these limitations by providing evolutionary context, detecting co-evolved gene sets, identifying contingent evolution and inferring functions of uncharacterized genes through comparative genomics [8,11,12]. A gene’s phylogenetic profile represents its conservation and loss patterns across a set of genomes [8]. PP is founded on the robust hypothesis that genes sharing similar phylogenetic profiles are likely to interact functionally [8,13,14,15]. Initially, PP employed binary scores to indicate the presence or absence of a gene across species [8,9,16]. As the number of genomes analyzed increased, more sophisticated approaches like normalized phylogenetic profiling (NPP) were developed to account for varying levels of conservation, reflecting cases where proteins diverge rather than disappear [13,14,17,18]. NPP employs a continuous conservation metric normalized by the phylogenetic distance from the query species (in our case, human), reflecting how similar the best-matching protein is to the human query after correcting for protein length and phylogenetic distance. This allows detection of gradual divergence rather than only binary presence or absence.

In recent years, we have shown that co-evolutionary signals can be highly complex and may vary across different parts of the tree of life [12,13]. To address this, we developed a “clade-wise” NPP approach that detects co-evolution within specific clades (groups of species sharing a common ancestor or segments of the evolutionary tree) and demonstrated that it can uncover functionally related genes. The “clade-wise” NPP approach has been successfully applied to uncover novel DNA repair genes [18,19] and identify potential therapeutic targets for MECP2 [20] and ACE2-associated disorders [21]. Moreover, a “guilt by association” approach enabled the discovery of disease-causing genes implicated in rare genetic diseases [22]. CladeOScope [17] was initially designed to enable users to investigate single genes, identifying co-evolved genes across all eukaryotes and within specific clades. Few tools currently offer comparable functionality [23,24,25]. Comparative genomics has traditionally relied on phylogenetic profiling to examine individual genes to infer evolutionary relationships and functional associations. However, biological processes are often orchestrated by groups of genes acting together, and the evolutionary history of these gene sets may not be fully captured by single-gene PP analyses or easily reconstructed from current databases of co-evolved gene clusters.

To address this gap, we developed a co-evolved Gene Set Analysis (GSA) tool, CladeOScope-GSA, focused on systematic evolutionary analysis of gene sets at different evolutionary scales. It enables users to evaluate the evolutionary cohesiveness of their gene lists, visualize conservation patterns across 1905 genomes, and identify clade-specific co-evolutionary behavior. The web tool is accessible at https://tabachlab.shinyapps.io/CladeOScope_staging/ (accessed on 26 January 2026).

## 2. Results

### 2.1. Gene Set Analysis (GSA)

**Figure 1:**
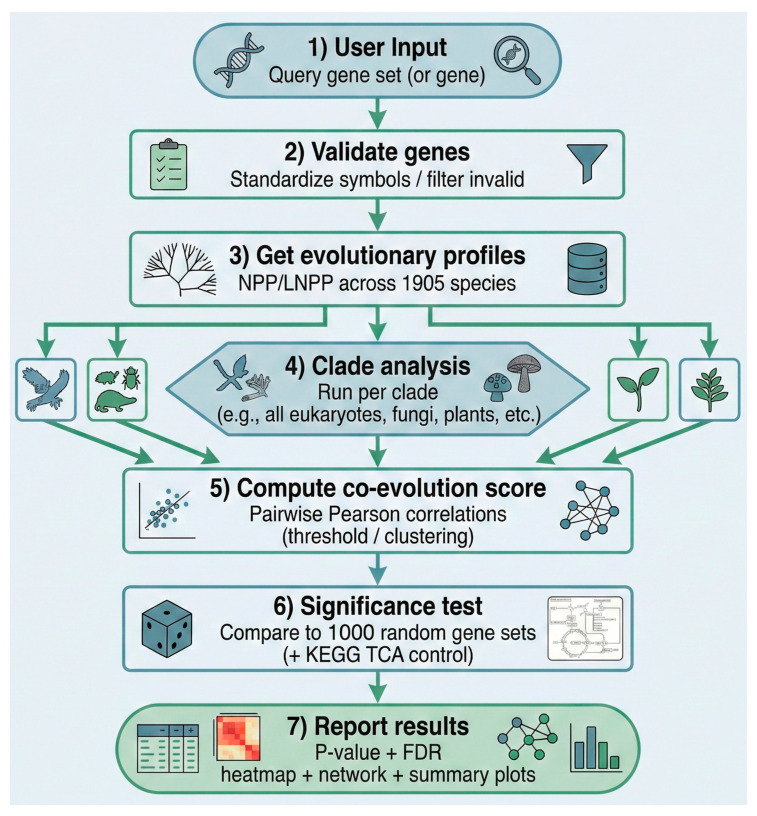
CladeOScope-GSA analysis workflow. Starting from a user-uploaded human gene set (HGNC symbols) and selected clades, CladeOScope-GSA maps genes to a precomputed normalized phylogenetic profiling (NPP) matrix (19,888 human genes × 1905 eukaryotic species), filters non-conserved genes, and retrieves clade-wise length-normalized phylogenetic profiles (LNPP). It then computes pairwise Pearson correlations across species to construct a correlation matrix, evaluates the co-evolutionary significance of the gene set by comparing threshold and cluster scores to 1000 size-matched random gene sets per clade, and applies hierarchical clustering and network construction (edges with correlation ≥ 0.7) to identify co-evolving clusters. The final outputs are heatmaps, clade-wise significance plots, co-evolution networks, and downloadable tables summarizing conservation and co-evolution metrics.

Gene set analysis is designed to evaluate phylogenetic correlations among genes in a user-defined set (Figure 1). Initially, CladeOScope-GSA assesses the co-evolutionary significance of the gene set using two distinct metrics (see Section 4). Upon submission, the query set is compared within each clade against 1000 pre-calculated random gene sets of the same size. This analysis provides users with insights into the degree of co-evolution within their input gene set. To enable users to examine and compare the evolution of the input genes and identify co-evolved clusters, CladeOScope-GSA generates both an NPP figure and correlation heatmaps.

### 2.2. Clade-Wise Analysis of Porphyria Genes Reveals Evolutionary Patterns Linked to Disease Etiology

**Figure 2:**
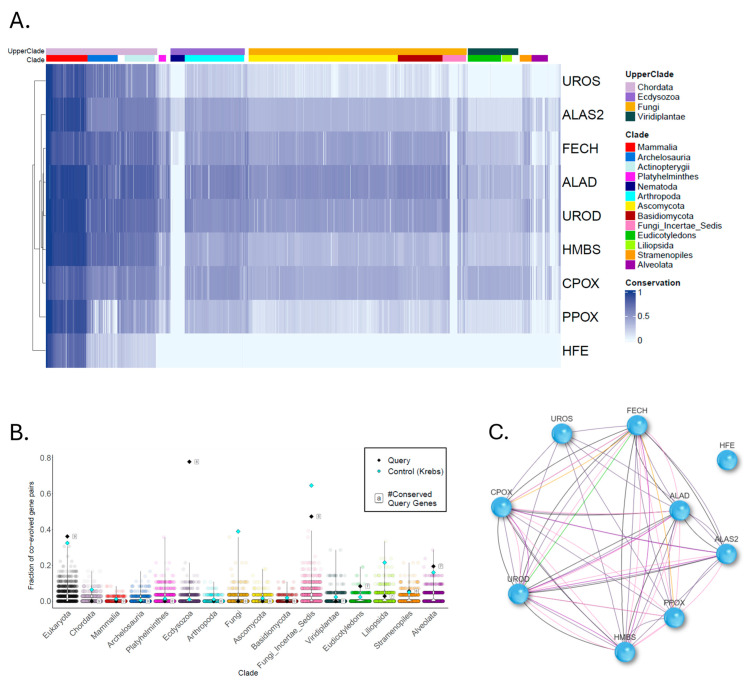
Evolutionary signatures of porphyria-related genes across 1905 eukaryotic species. (**A**) Heatmap showing the normalized phylogenetic profiles of nine heme-biosynthesis genes across the full species panel. Each row corresponds to a human query gene, and each column represents a species. Darker shades indicate higher conservation of the gene’s ortholog, whereas lighter shades denote reduced similarity or absence of an ortholog. (**B**) demonstrates how significantly co-evolved the query gene set is in eukaryotes, ecdysozoa, and fungi, in comparison to thousands of random sets of the same size, and to the Krebs cycle gene set. Each dot represents a set of random genes, colored by the clade in which the set was examined. The “#” symbol denotes the number of genes that are conserved in the corresponding clade and included in the analysis. Lastly, (**C**) the co-evolution network depicts pairwise gene-gene correlations above the defined threshold. Edge colors correspond to the clades displayed in the heatmap, indicating the clade(s) in which each gene pair shows significant co-evolution.

To demonstrate the ability of CladeOScope-GSA to provide new insights into gene lists analysis, we applied it to a well-studied gene set. Porphyria comprises metabolic disorders caused by mutations in heme biosynthesis genes, leading to enzyme deficiencies and toxic porphyrin accumulation [26]. Analysis of the nine porphyria-related genes reveals distinct conservation patterns (Figure 2). Nematodes and certain fungi, which are unable to synthesize heme de novo and must obtain it externally [27], exhibit low to absent conservation of these genes, leading to their distinct clustering. This co-evolutionary pattern is significant across eukaryotes, nematodes, and fungi (*p* < 0.0001; FDR < 0.0001), but not in chordates or other clades. Interestingly, while analysis of heme biosynthesis genes consistently identifies their co-evolution, the Homeostatic Iron Regulator (HFE) protein is notably absent from these clusters [14,25,28,29,30]. HFE displays a restricted phylogenetic profile, with detectable orthologs largely confined to vertebrates, and as such does not cluster with the porphyria-related heme biosynthesis genes. However, the absence of detectable HFE orthologs in more distant clades may also reflect limitations of BLAST-based (BLAST+ version 2.10.1) homology detection for rapidly evolving proteins, so an apparent ‘recent origin’ signal should be interpreted with caution.

The distinct phylogenetic profile (PP) of HFE suggests a role that differs from other heme biosynthesis pathway genes. HFE variants, associated with hereditary hemochromatosis, exacerbate iron overload, inhibiting uroporphyrinogen decarboxylase and thus triggering porphyria cutanea tarda [31]. This subtype of porphyria, which is indirectly linked to iron overload from HFE mutations, differs fundamentally from subtypes caused by direct enzyme defects. Our phylogenetic analysis was able to distinguish between these disease subtypes in agreement with clinical findings. This distinction helps explain why porphyria presents with such diverse clinical features and shows how gene set analysis can reveal functional groupings within disease pathways.

### 2.3. Phylogenetic Profiling of Dynein Gene Families Reveals Structural Subgroup Clustering

**Figure 3:**
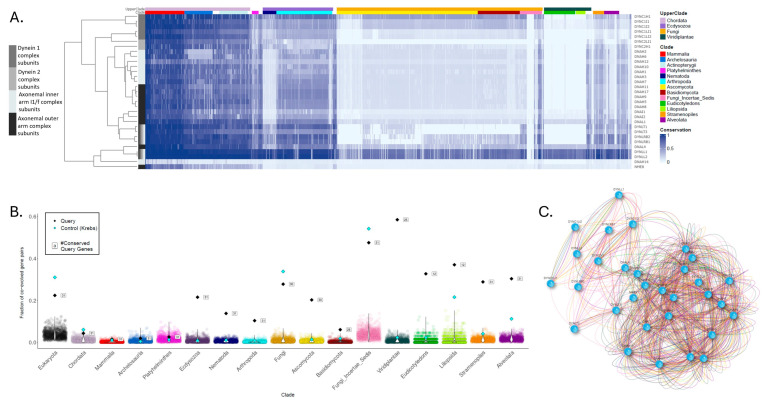
Clade-wise conservation patterns of the dynein gene family reveal a functional subgroup structure. (**A**) Heatmap of normalized phylogenetic profiles for human dynein genes across 1905 species. Rows represent individual dynein genes and columns species. Color intensity corresponds to the conservation level. The bar adjacent to the heatmap denotes each gene’s structural classification (e.g., cytoplasmic vs. axonemal). (**B**) illustrates the extent to which the query gene set is significantly co-evolved in eukaryotes, ecdysozoa, fungi, and plants, compared to thousands of randomly generated gene sets of the same size and to the Krebs cycle gene set. Each dot represents a random gene set, colored according to the clade in which it was analyzed. The “#” symbol represents the number of conserved genes within each clade. (**C**) The co-evolution network visualizes significant pairwise gene-gene correlations above the defined threshold. Edge colors indicate the clades shown in the heatmap, specifying the clade(s) in which significant co-evolution is observed for each gene pair.

The second example focuses on the dynein gene group (Figure 3). These genes were analyzed based on the dynein gene set as appears in the HGNC database [32]. The phylogenetic profile of dynein genes reveals distinct clustering corresponding to functional and structural subgroups. The heatmap highlights patterns of conservation across clades, with specific clustering observed among eukaryotes, ecdysozoa, fungi, and plants. The clade-wise analysis reveals that while cytoplasmic dyneins form a highly conserved module across the tree of life, several axonemal dyneins display restricted conservation, being retained in metazoans but lost in fungi and nematodes.

The evolutionary changes in dynein families reflect functional demands tied to cellular architecture and motility. In multicellular metazoans, axonemal dyneins are essential for ciliary and flagellar motility, underpinning processes such as left–right body axis determination, reproduction, and mucus clearance [33,34]. In contrast, fungi and nematodes, which either lack motile cilia/flagella or rely on alternative mechanisms for motility, have secondarily lost many axonemal dynein components [35,36]. By contrast, cytoplasmic dyneins, which drive intracellular transport along microtubules, remained indispensable in virtually all eukaryotic lineages due to their role in organelle positioning and cargo trafficking [37,38].

These patterns, consistent with structural data, offer evolutionary insights not accessible through individual gene profiling, supporting shared evolutionary pressures and structural roles among dynein gene families: the retention of cytoplasmic transport functions as a universal requirement, and the loss of axonemal motility functions in lineages where they became dispensable.

## 3. Discussion

Gene ontology and pathway analysis have become indispensable tools for understanding the complex functions of genes and their interactions within biological systems [1,39,40]. By providing structured frameworks for organizing biological knowledge, they enable researchers to interpret high-throughput data, discover functional relationships between genes, and gain insights into the mechanisms underlying normal physiology and disease [10,40,41].

CladeOScope-GSA introduces an additional evolutionary dimension to existing annotation frameworks. CladeOScope-GSA implements gene set evolutionary analysis and captures co-evolutionary signals and lineage-specific conservation patterns. Its statistical framework supports rigorous assessment of gene set enrichment or depletion across diverse taxa, facilitating insights into functional relationships, evolutionary constraints, and disease mechanisms. To uncover additional genes that co-evolved with each gene of a given subcluster, complementary single gene analysis can be performed.

By combining analytical power with a freely available, user-friendly, web-based interface, CladeOScope-GSA makes high-resolution phylogenomic analysis broadly accessible. Through these frameworks, researchers can move beyond studying individual genes in isolation to understanding how they function as part of interconnected systems, ultimately advancing our knowledge of biology and potentially leading to new therapeutic approaches for human disease.

## 4. Materials and Methods

### 4.1. Updated Database Content and Statistics

The normalized phylogenetic profiling (NPP) matrix was updated to include 19,888 human genes across 1905 eukaryotic species genomes, following a methodology similar to that previously described [13,14,17,21,25,42], with the following modifications:

#### 4.1.1. Genome Databases

The query Homo sapiens proteome was downloaded from UniProt proteomes on 17.03.2020. Reference whole proteomes were retrieved from three databases: Ensembl release 100, NCBI Genomes Refseq (August 2020), and Uniprot “reference proteomes” Release 2020_04. Proteome sequences for each species were merged, and duplicate sequences were filtered, resulting in a dataset of 1905 species.

#### 4.1.2. Gene Filtering

To normalize the matrix, each gene’s ortholog bitscore was divided by the bitscore of its human self-hit to account for protein length, creating a Length-Normalized Phylogenetic Profiling (LNPP) matrix. Genes with zero values across all species (43 in total) were excluded, resulting in a matrix of 20,355 genes and 1905 species.

The number of genes was further reduced to 19,888 to align with the GeneCards gene set. Gene symbols were standardized according to GeneCards nomenclature to enable seamless integration with its platform.

### 4.2. Evaluating Co-Evolution Significance: Threshold Score or Cluster Score

Threshold Score: The threshold score measures the percentage of pairwise Pearson correlations within a query gene set that exceed predefined thresholds (0.65–0.9). For a gene set of size N, the total number of gene-gene correlations is $\frac{n\left(n-1\right)}{2}$. CladeOScope computes the Pearson correlation (ranging from −1 to 1) and calculates the percentage surpassing the chosen threshold. While paralogous genes often exhibit correlations ≥ 0.9, and random genes around 0, non-paralogous genes that are known to be co-evolved may show values around 0.7. Therefore, a threshold value around 0.7 is recommended.

If the query set exceeds the size of the Krebs cycle gene set (used as a positive control according to KEGG, Tabach et al. [14]), r random genes are added to the control set to match its size. The additional genes are sampled uniformly at random from the same background pool of filtered human protein-coding genes, excluding genes already in the control set. This augmentation was used only to match set size for the threshold calculation, and results were stable across repeated random samplings. The Krebs cycle gene set was chosen as a positive control because previous normalized and clade-wise phylogenetic profiling studies have shown that it exhibits one of the strongest and most reproducible co-evolutionary signals among metabolic pathways across eukaryotes, driven by the sharp contrast between clades that retain a complete mitochondrial Krebs cycle and clades in which Krebs cycle enzymes are specifically lost or highly reduced while other mitochondrial functions remain conserved, making it an excellent benchmark for pathway-level phylogenetic cohesiveness.

Cluster score: In some gene sets, the input genes split into several clusters that do not correlate with one another. In these cases, the overall percentage of pairwise correlations above a chosen threshold may be low, even though distinct subgroups of genes clearly co-evolve. The cluster score is designed to be high when the genes show tight co evolution, represented by one dominant cluster or a few major clusters, and to decrease as more genes show uncorrelated evolution or multiple independent co evolution patterns. This metric is most informative for larger gene sets (typically >50 genes), where many genes do not co-evolve with each other.

To compute the cluster score, CladeOScope performs hierarchical clustering (complete linkage) of the gene set and “cuts” the resulting dendrogram at a height of 0.2 (corresponding to a minimal correlation of 0.8). At this cut height, the tool determines the number of clusters (i = 1…n) and their sizes, and uses these values to calculate the score:

$$\mathrm{Cluster}\;\mathrm{score}=\overset{n}{\underset{i=1}{\sum }}{\left(\frac{{\mathrm{cluster}\;\mathrm{size}}_{i}}{\#\mathrm{clusters}}\right)}^{2}$$

For each cluster i, “cluster size” is the number of genes in that cluster, and “#clusters” is the total number of independent clusters at height 0.2. Cluster sizes are squared to up-weight large coherent modules and penalize fragmentation into many small clusters, providing a single summary value of cluster cohesiveness. Non conserved genes are excluded from both the correlation heatmap and the random significance analysis.

### 4.3. Enhanced Web Interface

The website interface was upgraded to enhance usability and data visualization. Key improvements include detailed clade annotations displayed atop heatmap columns, improved color schemes and proportions in heatmaps and tables, and an interactive network representation of phylogenetic profile correlations, displaying interactions exceeding a threshold of 0.7. For downstream use, users can export plots directly from the interface in PNG or PDF format, and can also download the underlying phylogenetic profiles (NPP and LPP) as a CSV file. In addition, the inferred network (edge list) can be downloaded from the interface as a CSV file to support further analysis in external tools. Clade-wise analyses are performed using the predefined clade structure implemented in CladeOScope to ensure consistent and comparable results across runs. CladeOScope-GSA is available as a public web application (no registration required) and has been tested on recent versions of Chrome and Firefox. We currently support gene sets of up to 100 input genes per query, with typical runtimes ranging from under 1 min for small sets to 1–5 min for 100 genes, depending on server load.

### 4.4. Collaboration with the Genecards Suit

We recently launched a collaboration with GeneCards [43], enabling direct integration between the two platforms. Each gene in CladeOScope now links to its corresponding GeneCards page. Additionally, the GeneCards page for each gene lists its top 100 co-evolved genes across 17 clades and all eukaryotes, with direct links to relevant heatmaps on the CladeOScope website.

## Data Availability

All data used in this study are derived from publicly available genome and proteome resources, including UniProt, Ensembl, RefSeq, and GeneCards. The processed normalized phylogenetic profiling matrix and analysis scripts used for CladeOScope-GSA are available on GitHub (https://github.com/galgvili/CladeOScope-GSA/tree/main, accessed on 26 January 2026).
